# Efficacy of laser acupuncture for carpal tunnel syndrome: A study protocol for a prospective double-blind randomized controlled trial

**DOI:** 10.1097/MD.0000000000016516

**Published:** 2019-07-26

**Authors:** Chuan-Chih Chen, Yung-Tsan Wu, Yu-Chi Su, Yu-Ping Shen, Fang-Pey Chen

**Affiliations:** a Institute of Traditional Medicine, School of Medicine, National Yang-Ming University; b Department of Chinese Medicine, Tri-Service General Hospital, School of Medicine, National Defense Medical Center; c Department of Physical Medicine and Rehabilitation, Tri-Service General Hospital, School of Medicine, National Defense Medical Center; d Integrated Pain Management Center, Tri-Service General Hospital; e Department of Traditional Chinese Medicine, Taipei Veterans General Hospital, Taipei, Taiwan.

**Keywords:** carpal tunnel syndrome, laser acupuncture, traditional Chinese medicine

## Abstract

**Introduction:**

Carpal tunnel syndrome (CTS) is the most common entrapment neuropathy that causes hand discomfort and work disability. Since no satisfactory conventional treatments for mild to moderate CTS exist, we apply complementary alternative medicine (CAM) to this problem. Laser acupuncture (LA), a new, non-invasive therapy which uses low-level-laser therapy (LLLT) in acupuncture could help to manage CTS. However, only one small randomized, double-blind and crossover trial had been conducted, which is not enough to provide an evidence-based assessment of the effects of LA on CTS.

**Objectives:**

The aim of this study protocol is to investigate the efficacy of LA therapy on patients with mild to moderate CTS through sonography of the median nerve and offer clear parameters of LLLT.

**Methods:**

This study protocol is a prospective double-blind randomized controlled trial. Forty subjects aged 20 to 80 years old and diagnosed as having mild to moderate CTS will be randomly assigned to the intervention group (real LA, 3-sessions a week for 2 weeks) and control group (sham LA, 3-sessions a week for 2 weeks). All subjects will be asked to wear night splints as the fundamental management approach. The laser parameters will include a wavelength of 808 nm, power output of 300 mW and power density of 300 mW/mm^2^, with ten seconds of treatment for each acupuncture point (PC4, PC6, PC7, PC8, LI4, LI10, LI11, HT3, HT7, and LU10). Sham LA treatment will be applied without any laser power output. The primary outcome will be based the Boston Carpal Tunnel Syndrome Questionnaire and secondary outcomes included a visual analog scale, cross sectional area of median nerve by sonography and electrophysiological test before interventions and after 2, 4, 8, 12 weeks postintervention.

**Trial registration:**

ClinicalTrials.gov (Identifier: NCT03580265).

## 1 Introduction

Carpal tunnel syndrome (CTS) is the most common entrapment neuropathy resulting from compression of the median nerve (MN) as it passes through the carpal tunnel at the wrist.^[1]^ The prevalence of CTS is around 4% in Sweden^[2]^ and pain, numbness, or tingling in the thumbs, index, and middle fingers are the most common symptoms. Atrophy of the thenar muscle and weakness of pinch grasp may occur in patients with moderate to severe CTS.^[3]^ Hence, CTS often causes work disability.^[4]^ Although the pathophysiology of CTS is complicated, studies agree that persistent high pressure within the carpal tunnel compresses the MN and induces compression-related ischemia neuropathy.^[5]^

The treatments for CTS can be divided into conservative treatment and surgery. The conservative methods are suggested for mild to moderate CTS, including rest, nonsteroidal anti-inflammatory drugs (NSAIDs), splinting, corticosteroid injections, vitamin B, physical therapy, and so on.^[6,7]^ Although many conservative treatments are available, they fail to achieve satisfactory midterm and long-term effects.^[8]^ In addition, surgery is an invasive procedure with uncertain cost-effectiveness^[9]^; hence, surgery is only suggested for severe CTS or patients with unsatisfactory responses to conservative treatment. As a result, new conservative methods should be proposed to promote the success rates of treating mild to moderate CTS.

Low-level laser therapy (LLLT) is a non-invasive method and can promote wound healing, peripheral nerve regeneration, pain relief, and further reduction of inflammation.^[10]^ LLLT general applies wavelengths ranging from 600 to 1070 nm and power outputs ranging from 1 mW to 1000 mW.^[11]^ Some systematic reviews and meta-analysis studies have reported the significant efficacy of LLLT in different clinical conditions, such as temporomandibular disorders, fractures and plantar fasciitis.^[12–14]^ The therapeutic efficacy of LLLT for CTS was postulated in 1997–1998^[15,16]^ and studies have both investigated LLLT alone and compared it to other conservative treatments. Although most studies showed that LLLT leads to pain relief and hand function of CTS,^[17–22]^ a few studies found no significant benefits for CTS.^[23]^ A recent systematic review and meta-analysis revealed very short-term effects of LLLT for CTS.^[24]^ Lately, many clinicians have used LLLT on acupuncture points, which is called laser acupuncture (LA), to treat many clinical problems, such as musculoskeletal pain, lateral epicondylitis, headaches, etc.^[25]^ In contrast with traditional acupuncture needles, LA is a non-invasive therapy that does not cause tingling/pain during procedures.^[25,26]^

The application of LA for CTS is very rare and the first randomized, double-blind, placebo-control, crossover trial enrolled only 11 patients, as reported in a PubMed study in 2002.^[17]^ The result showed that patients with mild to moderate CTS experienced pain relief, improved Phalen's/Tinel's signs and sensory nerve conduction after LA therapy. However, the above study only investigated a few cases and applied a crossover design. Thus, the above findings do not confirm the definite therapeutic effect of LA for CTS. The purpose of our study aims to evaluate the clinical effect of LA for mild to moderate CTS through sonography of the MN and to offer clear parameters of LLLT.

## 2 Material and method

### 2.1 Ethics approval

The study protocol was approved by the Institutional Review Board of Tri-Service General Hospital (TSGHIRB: 1-107-05-062) and was registered with ClinicalTrials.gov (Identifier: NCT03580265). All recruited participants will be informed about the study procedure and provide informed consent before the study begins. The methods performed on each participant will follow the approved ethical guidelines. Personal information and study data will be collected and maintained in an independent closet and computer file with a password in order to protect personal data.

### 2.2 Study design

**Figure 1:**
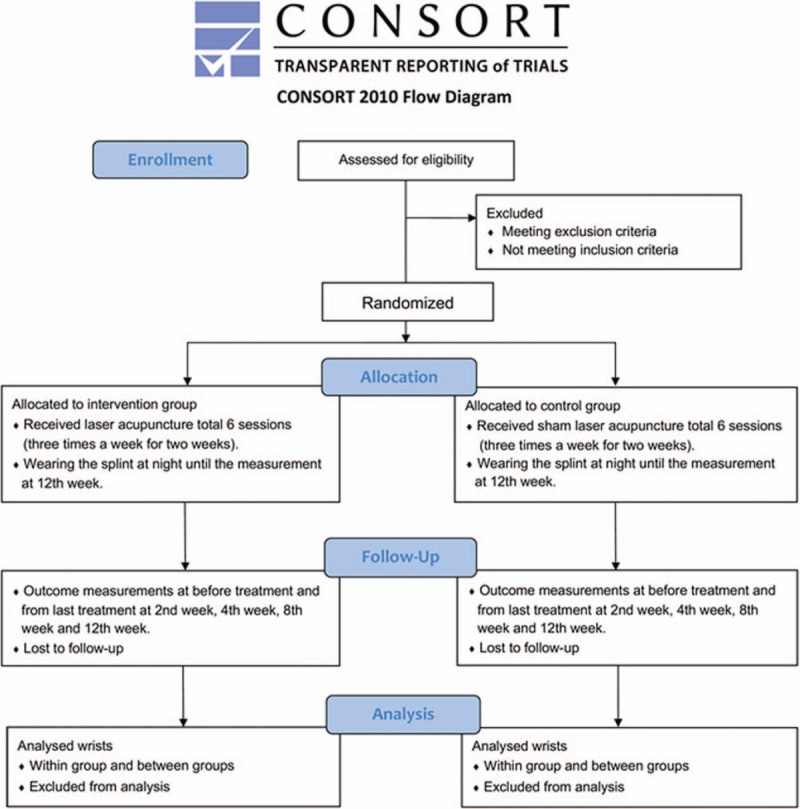
Study flow diagram.

**Figure 2:**
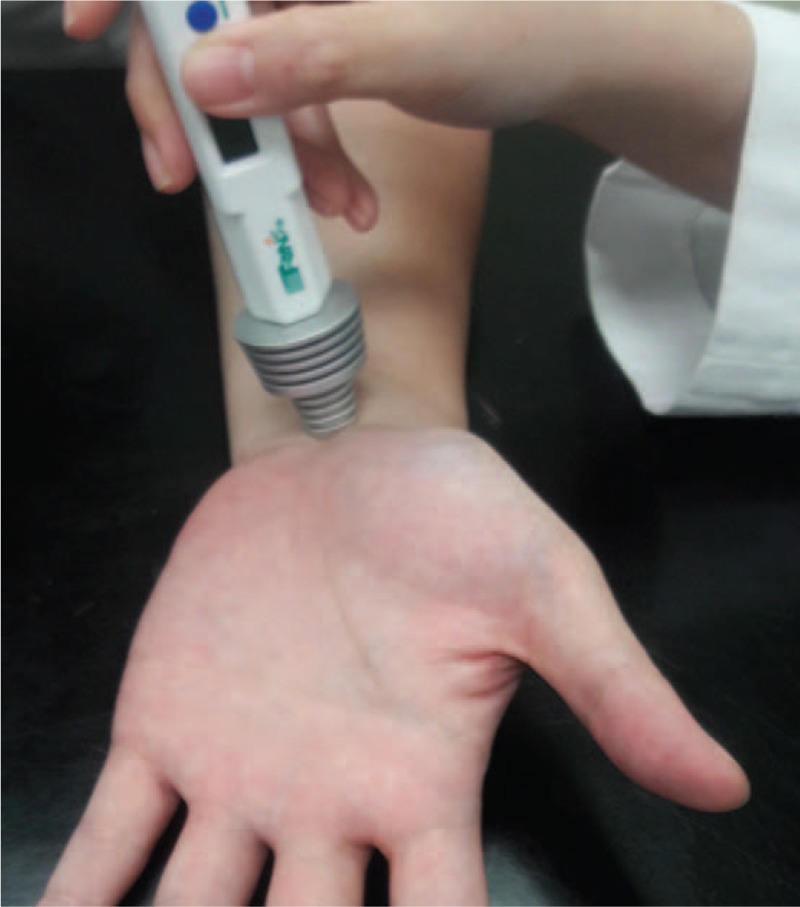
Laser acupuncture with device at the PC7.

**Figure 3:**
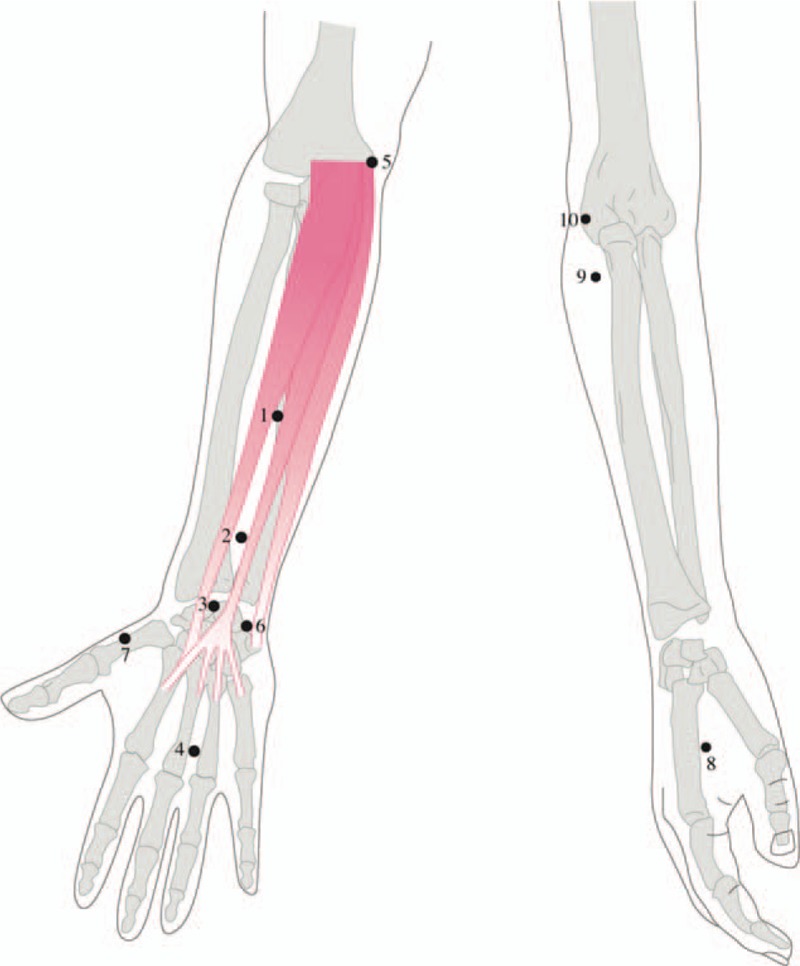
Acupoints used for carpal tunnel syndrome: 1. PC4, 2. PC6, 3. PC7, 4. PC8, 5. HT3, 6. HT7, 7. LU10, 8. LI4, 9. LI10, and 10. LI11.

This study protocol was designed as a prospective, randomized, double-blind, controlled trial according to the CONSORT 2010 statement (Fig. 1)^[27]^ and will be conducted at Tri-Service General Hospital in Taiwan in 2019. The recruited participants will be randomly assigned to the intervention group (real LA) or control group (sham LA) via computer-generated random numbers (1:1 ratio) using Microsoft Excel. If the patients are diagnosed with bilateral CTS, both wrists will be assigned to the same group. All subjects will wear night splints at least 8 h per night through the whole study period as the fundamental management approach.^[28]^ The LA device (“TRANS” Laser Phototherapy Device, TRANSVERSE INDUSTRIES CO., LTD, Taiwan) is like a pen (Fig. 2). A semiconductor was made of gallium–aluminum–arsenide (Ga–Al–As) with a wavelength of 808 nm, power output of 300 mW and power density of 300 mW/mm^2^ (following the guidelines of Jekins and Carroll).^[24,29]^ Real LA and sham LA will be applied by the same physician who is a licensed Chinese medicine practitioner in Taiwan. The ten acupuncture points include PC4 (Ximen), PC6 (Neiguan), PC7 (Daling), PC8 (Laogong), LI4 (Hegu), LI10 (Shousanli), LI11 (Quchi), HT3 (Shaohai), HT7 (Shenmen), LU10 (Yuji) (Fig. 2 and Fig. 3). Some of these points have used in other CTS studies.^[30,31]^ These acupoints are located according to the WHO Standardized Acupuncture Point Localization guidelines.^[32]^

### 2.3 Interventions

#### 2.3.1 Intervention group/real laser acupuncture group

Subjects in the intervention group will accept 3 sessions of real LA per week for 2 weeks (total 6 sessions). LA will be applied to each acupuncture point for 10 s with at least 4J/points based on the recommended LLLT treatment doses for CTS of the World Association of Laser Therapy (WALT).^[33]^

#### 2.3.2 Control group/sham laser acupuncture group

Subjects in the control group will accept the same number of sessions and interval times as the intervention group. Sham LA treatment will be applied without any laser power output. There will be no differences in observation, feeling, or listening between the two groups during the procedure. Hence, all patients in both groups will be blinded to group selection.

### 2.4 Participants

#### 2.4.1 Sample size

The necessary number of subjects required for our study is based on a previous study of the effects of LLLT for CTS.^[22]^ The sample size was calculated with a power of 80% (1 – β = 0.8), statistical significance (α = 0.05) of 95% and a dropout rate of 20%. As a result, this study will require at least 40 wrists.

#### 2.4.2 Inclusion and exclusion criteria

Participants aged 20 to 80 years diagnosed with mild- to -moderate CTS with clinical symptoms for at least 3 months and confirmed by electrophysiological testing will be enrolled. The definition of clinical symptoms/signs, and inclusion and exclusion criteria are summarized in Table 1. Patients will be excluded if they meet one of the exclusion criteria. The diagnosis and the grades of CTS are based on electrophysiological classification (Table 2).^[34,35]^

**Table 1:**
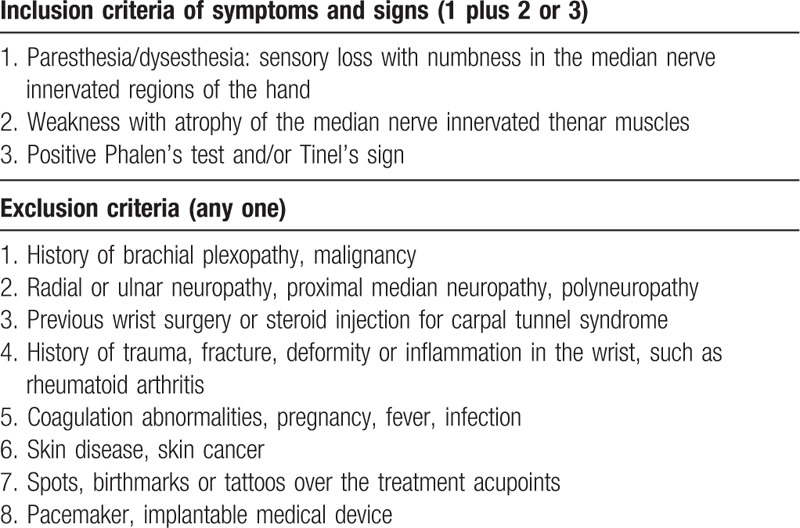
Summarization of inclusion and exclusion criteria.

**Table 2:**
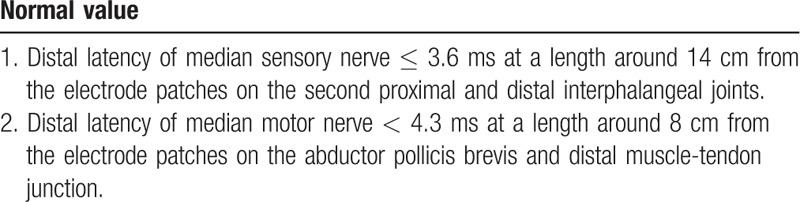
Electrophysiological classification of diagnosis and grades of carpal tunnel syndrome.

### 2.5 Outcome measurements

The same physiatrists who are blinded to the patients’ randomization will perform all the measurements for all participants of both groups before intervention and 2, 4, 8, and 12 weeks after intervention.

#### 2.5.1 Primary outcomes

##### 2.5.1.1 The Boston carpal tunnel questionnaire (BCTQ)

BCTQ is a disease-specific measurement of self-reported symptom severity and functional status for CTS.^[36]^ One is the minimum score and five is the maximum score on each symptom severity scale (SSS) (11 items) and functional status scale (FSS) (8 items). The total scores of SSS and FSS range from 11 to 55 and 8 to 40, respectively. We will use the Chinese version of the BCTQ, which had been corrected for reliability and validity.^[37]^

#### 2.5.2 Secondary outcomes

##### 2.5.2.1 Visual analog scale (VAS)

We will use VAS to measure the severity of digital pain or paresthesia/dysesthesia induced by CTS within 1 week before treatment and upon each follow-up time point.^[38]^ Zero is the minimum score for pain and ten is the maximum score for pain.^[39]^

##### 2.5.2.2 Electrophysiological test

Following the protocol, the electrophysiological test was developed by the American Academy of Neurology with SierraWave, Cadwell (USA).^[34]^ The setting of the active recording and stimulator is described in Table 2.^[40]^ Each measurement from the electrophysiological test will be made three times and averages of the values obtained will be determined for analysis.

##### 2.5.2.3 Ultrasonography of median nerve

Cross sectional area (CSA) of the MN at the proximal inlet of carpal tunnel was measured by using ultrasonography (MyLab^TM^ 25Glod, Esaote, Genova, Italy) in our previous study.^[40]^ CSA represents the swelling of MN with high validity for post-treatment evaluation.^[28,38,41]^ Each measurement from ultrasonography will be made three times and the averages of the values obtained will be calculated for analysis.

### 2.6 Statistical analysis

All statistical analyses will be performed with IBM SPSS Statistics Version 24. All data will be presented as mean ± standard error. The pair *t* test will be used to evaluate and compare before and after treatments within each study group. The independent *t* test and chi-square will be used to evaluate and compare before and after treatments between two groups. All statistical tests will be two-tailed and a *P*-value less than .05 is considered to be a statistically significant difference.

## 3 Discussion

To the best knowledge, the present study is the first prospective, randomized, double-blind, controlled trial to investigate the therapeutic effect of LA for mild to moderate CTS.

Previous studies indicated that LA therapy is a noninvasive technique with a painless, safe procedure and requires less time compared with needle-based acupuncture.^[42–44]^ Although the true effect of LA for CTS is uncertain based on a single small crossover study,^[17]^ current research of LLLT for CTS points to favorable short-term effects. A recent systematic review has shown that the effectiveness of LLLT can be maintained up to 5-weeks, but cannot be maintained upon 5 to 7-week follow-up. Moreover, reports of 3 to 6-month follow-ups provide limited evidence.^[24]^ Hence, a 3-month follow-up in our trial can adequately evaluate LA for CTS.

LA means applying LLLT to acupuncture points. Although the definite mechanism of LA is unclear, a combined effect of LLLT and traditional acupuncture is expected. In our opinion, LA not only requires less time than traditional LLLT, but also has a meridian treatment effect. The near infrared light from LLLT is absorbed by mitochondria, thus inducing the change of the cellular respiratory chain involving nitric oxide, reactive oxygen species and cyclic adenosine monophosphate (cAMP).^[10]^ One theory called the Acupoint Energy System states that acupoints are constituted by cells with more mitochondrial adenosine triphosphate (ATP).^[45]^ Therefore, the mechanism of LA seems to be associated with mitochondria, but further research is still needed.

In conclusion, LA is expected to be effective for the treatment of mild to moderate CTS and the results of this study will reveal its real benefit through sonography and electrophysiologic measurements.

## Acknowledgment

This work is particularly supported by Tri-Service General Hospital (TSGH-C108-128), Taipei Veterans General Hospital and the “Development and Construction Plan” of the School of Medicine, National Yang-Ming University (107F-M01-07M32).

## Author contributions

**Conceptualization:** Chuan-Chih Chen, Yung-Tsan Wu, Fang-Pey Chen.

**Data curation:** Chuan-Chih Chen, Yu-Chi Su, Yu-Ping Shen.

**Formal analysis:** Yung-Tsan Wu, Fang-Pey Chen.

**Investigation:** Chuan-Chih Chen, Yu-Chi Su, Yu-Ping Shen.

**Project administration:** Yung-Tsan Wu, Fang-Pey Chen.

**Supervision:** Yung-Tsan Wu, Fang-Pey Chen.

**Writing – original draft:** Chuan-Chih Chen.

**Writing – review & editing:** Yung-Tsan Wu, Fang-Pey Chen.
